# An endodermal subpopulation generates neural and mesodermal fates in the posterior chick embryo

**DOI:** 10.64898/2026.05.20.726401

**Published:** 2026-05-22

**Authors:** Panagiotis Oikonomou, Lisa Calvary, Devany Du, Juni Polansky, Giacomo Gattoni, Connor Lynch, Lingting Shi, Christian Mayer, José McFaline-Figueroa, Nandan L. Nerurkar

**Affiliations:** 1 Department of Biomedical Engineering, Columbia University; 2 Department of Biological Sciences, Columbia University; 3 Max Planck Institute for Biological Intelligence

**Keywords:** EMT, chick, endoderm, neuromesodermal progenitors, scRNAseq, lineage-tracing

## Abstract

The discovery of neuromesodermal progenitors (NMPs) — a bipotent progenitor population in the tailbud that gives rise to traditionally ectodermal and mesodermal tissues — has disrupted the classical view that progenitors of the three distinct germ layers are exclusively segregated during gastrulation. However, until now the notion of lineage restriction of the endoderm to ‘traditional’ gastrointestinal and respiratory tissues has largely remained intact. Here, we describe our discovery of a unique subpopulation in the chick endoderm that initially lines the ventral surface of the posterior organizer (Hensen’s node), but at the trunk-to-tail developmental switch, undergoes an FGF-dependent epithelial-to-mesenchymal transition, invading the tailbud and subsequently differentiating into a remarkably broad range of cell types including somites, notochord, and neural tube. Strikingly, ablation of this endodermal cell population results in a severe (~50%) reduction in axis elongation rate. Through single cell RNA sequencing and in situ hybridization chain reaction, we conclude that these cells lose their endodermal identity upon ingression, giving rise to NMPs that are biased toward mesodermal fates. Lineage tracing reveals that the node endoderm harbors a mixed multipotent population of progenitor cells capable of generating progeny that span endoderm and mesoderm or endoderm and ectoderm. These findings illustrate a previously unappreciated endodermal source of NMPs, and further demonstrates the breakdown of traditional lineage restriction of germ layers in the posterior embryo.

## INTRODUCTION

During embryonic development, one of the first major steps in lineage segregation is gastrulation, when a single totipotent layer of cells separates into three germ layers, endoderm, mesoderm, and ectoderm (Barresi & Gilbert, 2020). While conventionally, it was long accepted that germ layer commitment is exclusively established during gastrulation, this was upended with the discovery of neuromesodermal progenitor cells (NMPs), a cell population in the posterior embryo that persists after the end of gastrulation, and gives rise to both ectodermal and mesodermal cell types (Tzouanacou et al., 2009). NMPs derive from the posterior epiblast, ingressing into the tailbud mesenchyme as the primitive streak terminates at the end of gastrulation. Embryologically, NMPs occupy the tissue of the chordo-neural hinge, the region where the notochord and neural tube transition to the loose, disorganized mesenchyme of the tailbud (Henrique et al., 2015; Wymeersch et al., 2021). Molecularly, NMPs are generally defined by their co-expression of SOX2 and TBXT, classical markers of ectoderm and mesoderm, respectively (Wymeersch et al., 2021). During the past 17 years, NMPs have been rigorously studied to understand their embryonic origin (Garriock et al., 2015; Guillot et al., 2021) and the regulatory logic that drives their commitment to neural and mesodermal fates (Blassberg et al., 2022; Gouti et al., 2017; Kinney et al., 2020; Koch et al., 2017; Rito et al., 2025; Wymeersch et al., 2016).

Notably, there has been no prior evidence that NMPs contribute to endodermal tissues, or that in turn, the endoderm, which forms the ventral-most layer of amniote embryo, contributes to these dorsally-derived NMPs. Here, we describe an endodermal subpopulation in the posterior chick embryo that undergoes a previously unreported epithelial-to-mesenchymal transition, invading the adjacent tailbud mesenchyme from the ventral side and mixing with traditional, dorsally-derived NMPs. Disruption of cell ingression in this endodermal population significantly disrupts axis elongation, a phenomenon primarily attributed to mesodermal tissues (Bénazéraf et al., 2010; Xiong et al., 2020). Single cell RNA sequencing and gene expression analysis indicate that prior to ingression, these cells express endodermal markers, but down regulate them upon ingression as they increasingly adopt a broad range of fates. Through combined single cell barcoding and transcriptomics, we conclude that this endoderm population constitutes a mixed multipotent progenitor population that gives rise to cells spanning across traditional germ layer boundaries after gastrulation has ended.

## RESULTS

### Ventral node epithelium undergoes an epithelial-to-mesenchymal transition that begins at the trunk-to-tail transition

**Figure 1.**
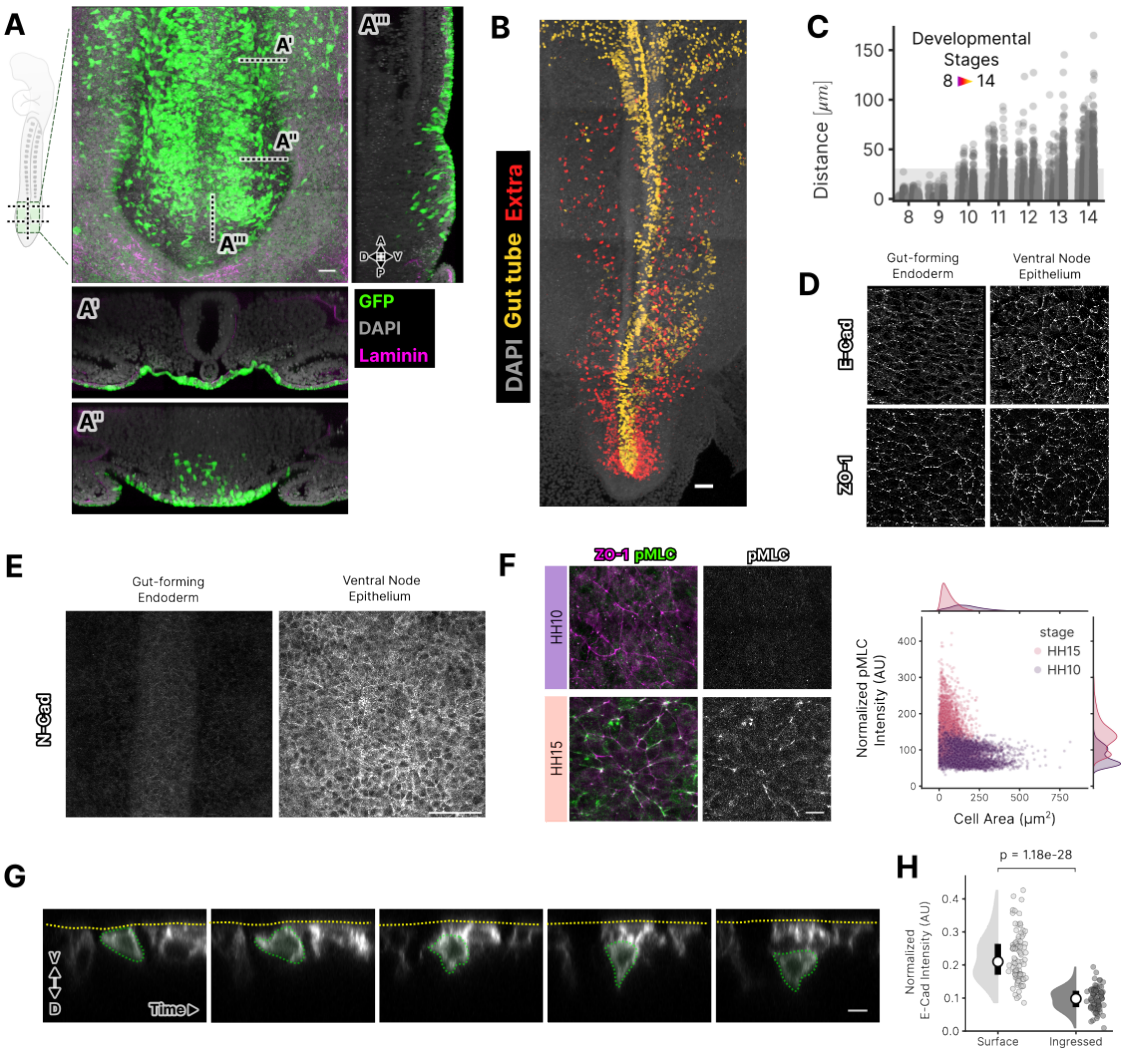
Cells of the ventral node ingress into the tailbud mesenchyme. (**A**) Ventral view of HH15 chick embryo following electroporation with a GFP reporter at HH11 with (A’) anterior transverse, (A”) posterior transverse, and (A”’) midline sagittal views as indicated; Scale = 30*μ*m. (**B**) Pseudocolored GFP+ cells (yellow: gut tube, red: extra) in HH18 embryo following electroporation of endoderm with GFP reporter at HH11; Scale = 50*μ*m. (**C**) Time series of cell ingression following electroporation with GFP reporter to tag node endoderm cells. Each dot is a a single cell, with alternating shades of grey indicating distinct embryos. Transparent grey bar indicates approximate basal position of surface cells, serving as a threshold beyond which cells are considered ingressed. (**D**) Immunostaining for E-cadherin and ZO-1 in gut forming (anterior to node) and node endoderm at HH15; Scale = 20*μ*m. (**E**) Immunostaining N-Cadherin in the gut-forming and node endoderm at HH15; Scale = 50*μ*m. (**F**) Immunostaining of apical ZO-1 and pMLC in the node endoderm prior to ingression (HH10) and after onset of ingression (left). Quantification of apical cell area and pMLC intensity at HH10 and HH15 (right). (**G**) Time-lapse stills from in vivo imaging of node endoderm during cell ingression (green annotation); apical surface of node epithelium annotated in dashed yellow; Scale = 10*μ*m. (**H**) Quantification of E-cadherin intensity from immunostianing, comparing surface node epithelium and ingressed cells.

To broadly perform fate mapping studies, we previously developed a high efficiency technique for electroporation-based transfection of the avian endoderm (Nerurkar et al., 2019). Through expression of a constitutive GFP reporter, we observed that throughout the embryo, expression is restricted to the endoderm, a convenient result of the electroporation method. However, surprisingly, at Hamburger Hamilton stage 15 (Hamburger & Hamilton, 1951), GFP-expressing cells can be seen outside the endoderm at the level of the tailbud, despite their restriction to the endodermal epithelium elsewhere in the embryo (Fig. 1A). By HH18 (approximately 72 hours of development), persistent GFP-expressing cells can be found distributed throughout axial tissues outside the primitive gut tube (Fig. 1B). A time course of fate mapping by electroporation (Fig. 1C) reveals that cells are retained within the ventral node epithelium until HH11, when they increasingly appear in the tailbud, suggesting that cells originating in the ventral node epithelium transition into the neighboring tailbud mesenchyme at the trunk-to-tail transition, when the mode of axis elongation switches from regression of Hensen’s node to outgrowth of the tailbud (Dias et al., 2020). Cells lining the ventral surface of the node express apically localized markers of polarized epithelia, including ZO-1 and E-Cadherin, but are less organized than gut forming endoderm (Fig. 1D). Therefore, the ventral node population has an epithelial identity. However, by HH15, when ingression is underway, these cells are enriched for N-Cadherin when compared to gut forming endoderm (Fig. 1E), and upregulate apically localized phospho Myosin Light Chain (pMLC, Fig. 1F). Within the ventral node epithelium, cells with smaller areas are enriched for pMLC, consistent with apical constriction (Fig. 1F). Live imaging reveals ingression of cells from the ventral surface of the node (Fig. 1G, Supp. Movie 1). Finally, ingressed GFP+ cells downregulate E-Cadherin, consistent with loss of epithelial identity (Fig. 1H). Together, these data suggest that the ventral node epithelium undergoes an epithelial-to-mesenchymal transition that initiates at the trunk-to-tail transition.

### The ventral node epithelium is endoderm

**Figure 2.**
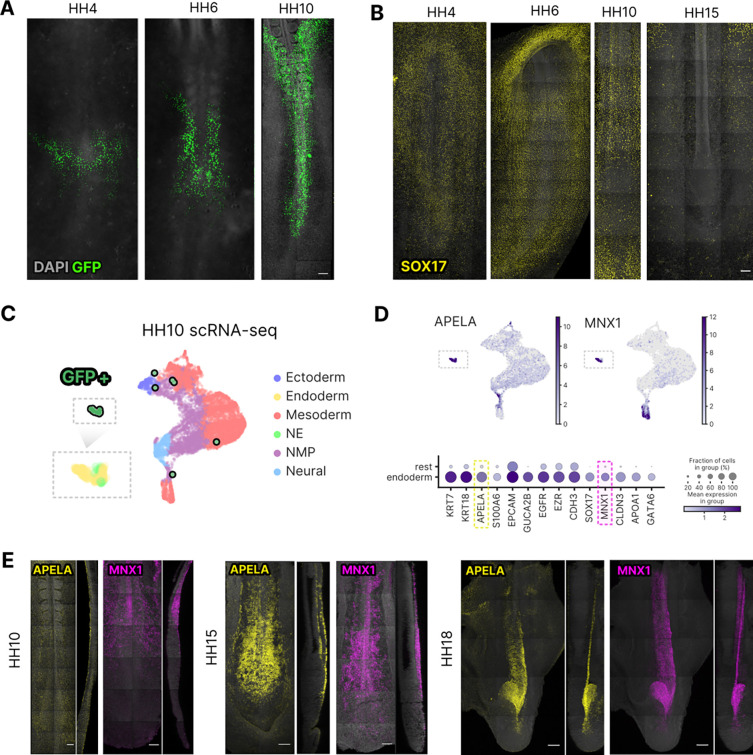
The ventral node epithelium is an endodermal tissue. (**A**) Fate mapping by RNA electroporation to tag node epithelium during gastrulation at HH4, with GFP-expressing node epithelium displacing posteriorly with the node during axis elongation to HH10; Scale = 100*μ*m. (**B**) Immunostaining for classical endoderm marker Sox17; Scale = 50*μ*m (**C**) Dimensionality-reduced 2D embedding of scRNA-seq dataset generated from posterior tissue of HH10 embryos following electroporation with GFP reporter plasmid to tag node epithelium; cells in which GFP transcripts were detected are indicated in dark green. (**D**) Identification of endoderm markers as differentially expressed genes between the endoderm cluster in (C) and all other cell types. (**E**) APELA and MNX1 in situ HCR across stages to validate their use as endodermal markers; Scale = 100*μ*m.

Prior fate mapping studies demonstrated that the ventral node consists of definitive endoderm, cells that ingress early through the anterior primitive streak during gastrulation and re-epithelialize at the ventral surface of the embryo (Lawson & Schoenwolf, 2003). We performed fate mapping by RNA electroporation to tag the ventral node epithelium at HH4, observing that these cells descend with the node as it regresses during gastrulation (Fig. 2A). These cells transiently express the conventional endoderm marker SOX17, but together with much of the posterior gut forming endoderm, down regulate SOX17 expression as development proceeds (Fig. 2B). To definitively test the identity of ventral node epithelium prior to the onset of ingression, we performed single cell RNA sequencing following electroporation with a constitutive GFP reporter to tag the ventral node epithelium. Detection of GFP transcripts was used to identify these cells within the dataset (Fig. 2C). We found that GFP-expressing cells were tightly clustered with endoderm, reflecting a transcriptional state most consistent with definitive endoderm (Fig. 2C). In situ hybridization chain reaction (HCR) for additional endodermal markers APELA (Lozovska et al., 2023) and MNX1 (K.-R. Li et al., 2025; L.-C. Li et al., 2021) at HH15 and HH18, when SOX17 expression is undetectable in the posterior endoderm, revealed that ventral node epithelium highly expresses both markers persistently (Fig. 2D, E). For both APELA and MNX1, we observed some low level expression subjacent to the node epithelium, coinciding with the site of cell ingression (Fig. 2E). Together, these data suggest that the ventral node epithelium has an endodermal identity prior to ingression. From here forward, we refer to this cell population as node endoderm (NE).

### Node endoderm (NE) give rise to neuromesodermal progenitor cells (NMPs)

**Figure 3.**
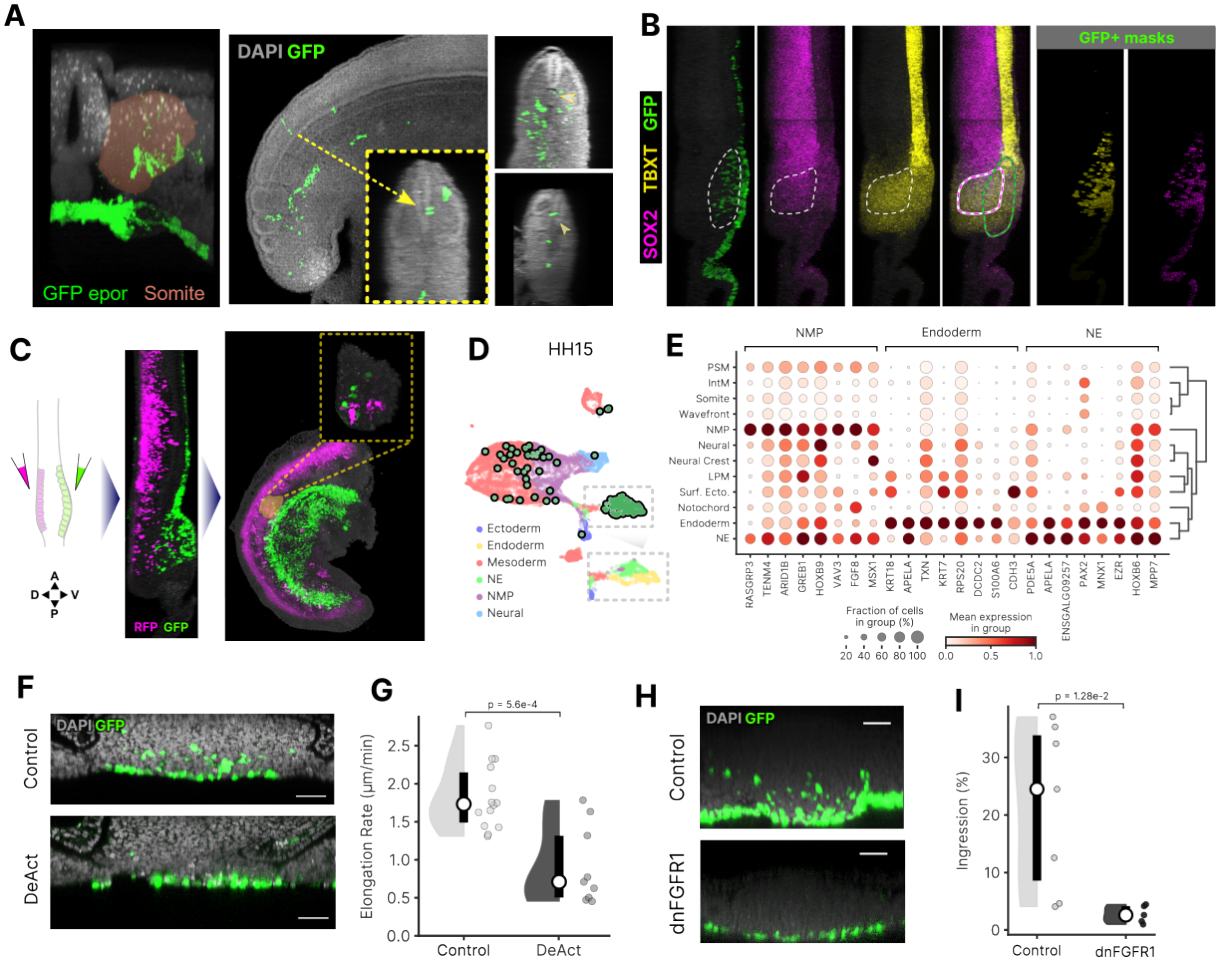
Node Endoderm gives rise to Neuromesodermal Progenitors. (**A**) Embryonic day 4 embryo following sparse in ovo labeling of endoderm via electroporation, with GFP+ cells incorporated into somites (left), neural tube and notochord cells (center/right). (**B**) HH15 embryos following electroporation to tag GFP+ endoderm with multiplexed in situ HCR for TBXT/SOX2, green dashed line marking the region of ingressing cells and magenta dashed line marking the region of TBXT+/SOX2+ coexpression. GFP+ mask (right) highlights co-expression of TBXT and SOX2 in ingressed node endoderm descendants. (**C**) Dual electroporation approach to differentially tag ventral (endoderm) and dorsal (epiblast) ingressing cells with GFP and RFP, respectively. Inset reveals isolated somite in which both ventral and dorsal ingressing cells colocalize. (**D**) Dimensionality-reduced 2D embedding of scRNA-seq dataset from HH15 embryos with endoderm tagged via electroporation with GFP reporter; Cells in which GFP transcripts were detected are indicated by dark green circles. (**E**) Differentially expressed genes comparing NMP, NE and Endoderm clusters. (**F**) Transverse view of HH15 embryos following targeted electroporation with DeAct-GFP construct to disrupt actin cytoskeleton, effectively ablating node endoderm and blocking ingression. (**G**) Quantification of axis elongation rate between GFP (control) and DeAct-GFP electroporated embryos. (**H, I**) Dominant negative (dn) FGFR1 electroporation disrupts ingression compared to GFP alone, quantified in (I). PSM: Presomitic Mesoderm, IntM: Intermediate Mesoderm, NMP: classical neuromesodermal progenitor, LPM: Lateral Plate Mesoderm, NE: Node Endoderm.

To determine the fate of NE cells upon ingression, we performed fate mapping experiments by tagging the endoderm via electroporation with a GFP reporter at the onset of ingression at HH11, and developing embryos to later stages. We observed GFP-expressing descendants of the endoderm distributed across tailbud mesenchyme and inserted into mesodermal and ectodermal structures, including somites, notochord, lateral plate mesoderm, neural tube, and surface ectoderm (Fig. 3A). Mesodermal contributions were qualitatively more frequently observed than ectoderm. Based on these findings, we considered whether the NE may be a previously unrecognized source of NMPs. While the true definition of NMPs has been contested in the literature (Edri et al., 2019; Kondoh & Takemoto, 2024; Morabito et al., 2025; Wood et al., 2019), coexpression of ectodermal marker SOX2 and mesodermal marker TBXT is most widely accepted as the hallmark of NMPs (Gouti et al., 2017; Rito et al., 2025; Wymeersch et al., 2021). By HCR, we observed that GFP-tagged NE-descendants co-express SOX2 and TBXT, suggesting that they are indeed NMPs (Fig. 3A). When dual-labeling experiments were performed, targeting expression of GFP to the ventral NE cells and RFP to the dorsal cell population that gives rise to traditional NMPs, we found that both cells occupy the chordo-neural hinge by HH15 (Fig. 3C), and at later stages, both NE descendants and traditional NMPs can be observed incorporating into mature somites. To test the identity of ingressed NE descendants more rigorously, we again turned to single cell RNA sequencing following tagging of endoderm cells via expression of a constitutive GFP reporter. Unlike HH10, when GFP-expressing cells were largely restricted to the endoderm, at HH15 we observed a broader distribution of GFP+ cells, including colocalization with NMP and mesoderm clusters (Fig. 3D).

Recognizing that NE behaves as a distinct subpopulation within the endoderm, we isolated endodermal subclusters within the single cell dataset, identifying 3 distinct subpopulations. Through HCR against differentially expressed genes between these clusters, we identified a BAMBI+ subpopulation that lies posterior to the node region, extending antero-laterally around the central gut-forming endoderm in a U shape (Fig. S1). Gut forming endoderm and node endoderm co-express several genes, including APELA and MNX1 (Fig. 2, S1). This is consistent with the identity of node endoderm as definitive endoderm despite no known role in gut tube formation. Nonetheless, we identified a handful of genes that are expressed throughout the node, including node endoderm, which were not detected in the gut forming endoderm, including FGF8, FGF19, and MSX1. Returning to the full single cell dataset representing the posterior HH15 embryo, hierarchical clustering revealed that node endoderm most closely aligns with gut forming endoderm, but also highly expresses NMP-specific genes (Fig. 3E). Therefore, based on embryological observations (node endoderm cells populate the chordoneural hinge), fate mapping (node endoderm contributes to mesodermal, and to a lesser extent ectodermal, structures) and transcriptional states, we conclude that node endoderm cells are a previously unappreciated source of NMPs.

To test functionally the importance of node endoderm as an NMP source, we first broadly disrupted the cell population by targeted electroporation to express DeAct, a construct that severely disrupts the actin cytoskeleton (Harterink et al., 2017; Oikonomou et al., 2025; Powell et al., 2025). In addition to effectively blocking cell ingression, DeAct led to delamination and cell death within the node endoderm, effectively functioning as a method to selectively ablate the node endoderm (Fig. 3 F). Surprisingly, this resulted in a ~50% reduction in axis elongation (Fig. 3G), a behavior primarily attributed to mesodermal tissues in the chick embryo (Bénazéraf et al., 2010; Xiong et al., 2020). To test this with a more gentle and targeted approach, we inhibited FGF signaling in the endoderm via misexpression of a dominant negative FGFR1 mutant. FGF signaling orchestrates a range of processes in the posterior embryo, and has also been implicated in cadherin switching and EMT. Indeed, dnFGFR1 over expression in the node endoderm dramatically reduced cell ingression (Fig 3H,I). Together, these results suggest that node endoderm plays a significant role in axis elongation, although it is not clear whether this is directly as a source of mesodermal cells that expand the antero-posterior axis, or indirectly via cell-cell communication or other interactions.

### The node endoderm is a mixed multipotent progenitor population that gives rise to clonal populations across germ layers

**Figure 4.**
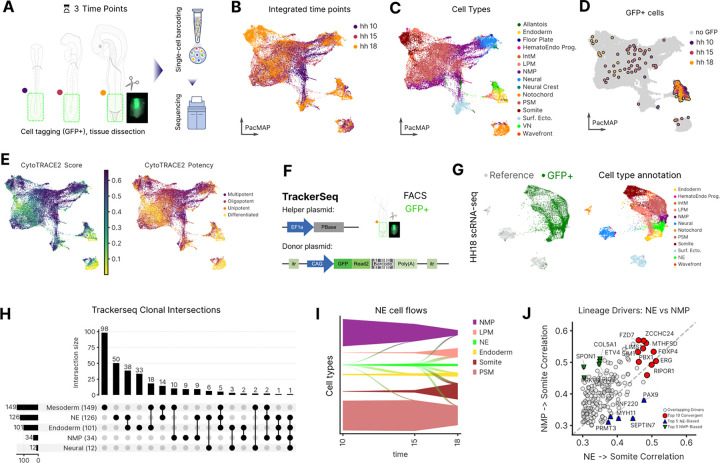
Single cell RNA sequencing, lineage tracing and trajectory inference. (**A**) multi-timepoint scRNAseq experimental setup. (**B**) Batch-integrated time points in shared embedding. (**C**) Cell type annotations of the developing chick tailbud. (**D**) Cells with detectable GFP transcripts (endoderm descendants), color coded by stage. (**E**) Cytotrace2 potency score, absolute continuous scale (1 totipotent, 0 terminally differentiated) and categorical potency label. (**F**) Trackerseq plasmid and experimental setup. (**G**) FACS enriched GFP-expressing cells (endoderm descendants) registered against the HH18 single cell dataset for cell type identification (left), and cell cluster identities of the HH18 dataset (right). (**H**) UpSet plot showing lineage relationships for populations of interest. (**I**) Cellular flows of node endoderm cells inferred by optimal transport between time points. (**J**) Comparison of lineage drivers between two converging differentiation trajectories, classical NMPs and node endoderm, towards somitic fate. Red data points indicate shared drivers of somite formation, blue/green triangles indicate drivers associated with NMP/node endoderm.

Recognizing that the node endoderm represents a progenitor population giving rise to mesodermal, and to some extent ectodermal cell types, we next interrogated the lineage trajectories of this population through inference on single cell datasets (Fig 4A). Batch integration across three developmental stages yields an embedding with smooth transitions across developmental stages (Fig 4B). Isolating GFP+ cells across time points confirmed diversification of endoderm descendants from HH10 to HH18, although many cells retained their endodermal identity at HH18, including enrichment of both gut forming and node endoderm cells types (Fig 4C, D). Multipotency was estimated from the full time-integrated dataset spanning HH10 (prior to onset of ingression), HH15 (ingression is well underway), and HH18 (the latest time points accessible in EC culture of chick embryos). To do so, we adopted Cytotrace2, a deep learning tool trained on diverse developmental scRNAseq datasets to identify developmental potential and differentiation landscape in an unbiased way (Kang et al., 2025). Cytotrace2 computed potency scores revealed a complex mixture of differentiation potential across the posterior chick embryo, and assigning cell states as either differentiated or multi, oligo, or unipotent revealed that the transcriptional state of node endoderm is most consistent with a multipotent progenitor population (Fig 4E). This was despite the fact that the node endoderm cluster was identified on the basis of marker gene expression that do not show any obvious overlap with pluripotency genes, which drive Cytotrace2 cell classification.

While inference tools such as Cytotrace2 can be highly informative for interrogating putative differentiation states from existing datasets, true lineage requires tracking heritable signatures across progeny of single cells. To do this, we turned to Trackerseq, a lineage barcoding technique compatible with single cell RNA sequencing, so that lineage and cell states can be simultaneously determined (Bandler et al., 2022). In brief, the method relies on genomic integration of barcodes downstream of GFP and a constitutive promoter (CAG), so that lineage signatures are detectable within the transcriptome. The endoderm was coelectroporated at HH10 with a plasmid library containing up to 125 million unique barcode sequences together with a helper plasmid containing transposase under control of the EF1*α* constitutive promoter. At HH18, tailbuds were dissociated and FACS enriched for GFP+ cells which comprise descendants of the endoderm that carry lineage barcodes. Cell states were determined by registering these cells against the full HH18 dataset (Fig. 4G), which contains both endodermal and non-endodermal cells of the posterior chick embryo. Because this dataset was enriched for endodermal descendants by FACS, we observed a remarkably broad range of cell identities, spanning across traditional germ layer boundaries. Consistent with our prior findings (Fig. 3D), endoderm derived cells were most enriched within endodermal fates along with paraxial and lateral plate mesoderm (Fig. 4G, H); however we also noted endoderm descendants within the notochord and both surface and neural ectoderm 4G, H).

Turning to clonality of these various cell types, we found that the most abundant clonal populations were restricted to mesoderm, suggesting that the node endoderm likely contains a large proportion of unipotent progenitors fated to give rise to mesodermal cell types even prior to ingressing from the node endoderm (Fig. 4H). The next largest number of clones retained a node endoderm identity, suggesting that a significant proportion of node endoderm cells are self-renewing between HH10 and HH18. Importantly however, several clonal populations panned multiple cell types, even crossing conventional germ layer boundaries (Fig. 4H). Clonal populations were detected that spanned node endoderm and either endoderm, mesoderm, NMPs, or ectoderm (though this fraction was low). These findings further support the existence of fate restricted progenitors in the node endoderm that are capable of self renewal and differentiation into one of the three germ layers. However, we also observed clonal populations that spanned endoderm and mesoderm - but not node endoderm - supporting the existence of bipotent mesendoderm like cells within the node endoderm. Taken together, these data indicate that the node endoderm is a mixed multipotent progenitor population containing fate restricted and bipotent progenitor cells.

### Comparison of inferred lineage drivers for paraxial mesoderm differentiation from node endoderm and NMPs suggests distinct but overlapping paths to somitogenesis

Our findings suggest that the node endoderm is a previously unappreciated source of NMPs in the avian tail, with a particularly important contribution to the paraxial mesoderm arising through ingression at the ventral surface of the embryo. However, to date, studies of axis elongation and somitogenesis in the chick embryo have focused only on mesoderm that is generated directly from the epiblast, ingressing from the dorsal side of the embryo during gastrulation (Guillot et al., 2021). We next relied on trajectory inference to identify putative lineage drivers for somitogenesis from either node endoderm or NMPs as a progenitor state. To do so, we returned to the single cell RNA-Seq time course, first employing Optimal Transport (Klein et al., 2025; Weiler et al., 2024) to reconstruct cell flows across HH10, HH15, and HH18 (Fig. 4I). Taken together with Cytotrace2 potency scores, we then constructed inferred differentiation trajectories, finding that both node endoderm and conventional NMPs give rise to somites (Fig. 4I). Computing putative lineage drivers of each, we isolated many genes implicated in somitogenesis, including FOXC2 (Kume et al., 2001), FZD7 (Borello et al., 1999), PBX1 (Capellini et al., 2008), that are shared by both NMP and node endoderm cells differentiating into somites (Fig. 4J). We also identified genes that have — to our knowledge — not previously been implicated in somitogenesis or paraxial mesoderm differentiation, including FOXP4 and ZCCHC24. It is possible that the role of these genes in somitogenesis have been obscured in previous studies that focused only on direct mesodermal contributions to somitogenesis. Interestingly, we also identified divergent lineage drivers that were specific to either node endoderm or NMPs (Fig. 4J), suggesting that somitic mesoderm may be produced by fate convergence from two disparate progenitor populations following distinct initial trajectories.

## DISCUSSION

The present study reveals a previously unappreciated source of multipotent progenitor cells in the posterior endoderm that give rise to diverse cell types across traditional germ layer boundaries. These cells undergo EMT from the ventral surface of the tailbud (Fig. 1), losing their initial endodermal identity upon ingression (Fig. 2), and giving rise to cells that adopt fates as diverse as lateral plate mesoderm, and neural ectoderm, among others (Fig. 3–4). These node endoderm cells are distinct from traditional NMPs because they also give rise to gut-forming endoderm, though it is inconclusive whether single cells within this population can give rise to cells of all three germ layers. Nonetheless, the present study further illustrates the breakdown of traditional lineage segregation along germ layers in the posterior vertebrate embryo.

The existence of mesendoderm cells in the amniote embryo has been disputed in the literature, with some arguing that a persistent population in the epiblast is capable of generating endodermal and mesodermal cells (Masamsetti et al., 2025), while others suggest that these are fate restricted endoderm and mesoderm progenitors that co-localize within the epiblast, and any true mesendodermal state is brief and transient (Mittnenzweig et al., 2021; Probst et al., 2021). Here, we find through lineage barcoding that node endoderm does contain true ‘mesendoderm’ cells capable of generating endodermal and mesodermal fates. However, these cells are distinct from the conventional notion of amniote mesendoderm, because they arise from endodermal epithelium after gastrulation has ended, they occasionally produce ectodermal cells, and at earlier stages appear to be firmly of endodermal identity based on their aggregate transcriptional state (Fig. 2C). Nonetheless, the present study does support the existence of mesendodermal cells, and warrants revisiting lineage segregation during gastrulation. In future work, it may be insightful to ask to what extent ingression of node endoderm constitutes re-initiation of a gastrulation-like program.

Despite the broad range of cell types generated from node endoderm, our lineage barcoding experiments failed to identify significant clonal populations that span all three germ layers. Whether such a population exists is inconclusive, owing in part to limitations in the present study. The initial observation that node endoderm ingresses was only possible because of high efficiency electroporation of the endoderm, which requires removal of the chick embryo from the egg, followed by ex ovo culture using the well established EC method (Chapman et al., 2001). However, all experiments in the present work have HH18 as the terminal time point because this is the latest stage to which embryos can be cultured using this method. At HH18, however, we find by Cytotrace2 that many node endoderm descendants retain multipotency, and by Trackerseq we confirm that several clonal populations retain node endoderm identities. This suggests that at HH18, progenitors within node endoderm are not yet exhausted, and it is possible that longer term lineage tracing experiments may reveal a broader range of clonally linked cell fates.

The present study suggests that many classical aspects of vertebrate development may need to be revisited or reframed. For example, endoderm has largely been considered to give rise to respiratory, gastrointestinal, and endocrine tissues, with some small exceptions (Larkins et al., 2026). However, we find that node endoderm gives rise to somites and lateral plate mesoderm, suggesting that endoderm may give rise to cells of the musculoskeletal system as well. The dramatic effects of node endoderm ablation on axis elongation suggest that this contribution may be substantial within the posterior embryo. Second, despite extensive study, the definition of NMPs is contested, with some suggesting that SOX2/TBXT coexpression alone is insufficient to mark NMPs (Edri et al., 2019; Morabito et al., 2025; Mugele et al., 2018). One possible source of disparity in the literature may be that SOX2/TBXT double positive cells include both endoderm derived NMPs and conventional NMPs, which based on our transcriptional analyses are similar but not identical. With recent expansion of organoid and in vitro directed differentiation models for NMP biology, there appears to be a strong underlying bias of NMPs in vitro to adopt a neural rather than mesodermal fate (Edri et al., 2019; Wang et al., 2026). Given that node endoderm descendents are strongly biased toward mesodermal fates, recognizing the disparate embryonic sources of NMP subpopulations may provide new insight into the defining feature of NMPs, and what guides their differentiation along distinct neuromesodermal lineages.

## METHODS

### Chicken embryology techniques, electroporations

Fertilized White Leghorn chicken (Gallus gallus domesticus) eggs were acquired from University of Connecticut Poultry Farm and incubated at 37° C and 60% humidity for 43 hours (HH stage 11). Embryos were harvested onto filter paper rings and transferred to plates containing EC culture medium (Chapman et al., 2001). Endoderm-specific electroporation was carried out as previously described (Nerurkar et al., 2019; Oikonomou et al., 2025). Briefly, electroporation solutions were prepared by diluting pCAG-EGFP, pCAG-myr-mRFP (Addgene #32604), or pCAG-DeAct-SpvB (a fusion protein of DeAct-SpvB with EGFP, subcloned into pCAG from Addgene #89446) plasmids to 3.5 *μ*g/*μ*L in molecular grade ddH2O with 5% sucrose and 0.1% Fast Green FCF. Following delivery of the DNA solution to the ventral surface by micropipette, embryos were electroporated using a Nepa 21 transfection system (Nepa Gene, Ichikawa City, Japan) with a sequence consisting of three 35V poring pulses of 0.2 msec duration separated by 50 msec with a decay rate of 10% between successive pulses, followed by five 4V transfer pulses of 5.0 msec duration separated by 50.0 msec with a 40% decay rate (Nerurkar et al., 2019; Oikonomou et al., 2025). Linearized pCS2–3nls-EGFP plasmid (Addgene #165400) was used for in vitro transcription of mRNA. 500 *μ*g*μ*L mRNA with 10 % sucrose, and 0.1 % fast green in RNAse-free ddH2O was electroporated as previously described (Powell et al., 2025).

### Immunofluorescence and in situ Hybridzation Chain Reaction (HCR)

Embryos were fixed overnight in 4% paraformaldehyde in PBS, rinsed, and dissected from surrounding extraembryonic tissue and filter paper. Embryos were washed (PBS with 0.5% Triton was used for all washes) and left in blocking solution (10% heat inactivated goat serum) for 2 hours, then incubated overnight at 4° C with primary antibody diluted in pre-block buffer. After five short 5 min washes, embryos were incubated secondary antibodies and DAPI (1:1000). Embryos were then cleared in RapiClear (RapiClear 1.49, Sunjin Labs) and mounted on glass slides for imaging. Specific protocols per antibody: Fibronectin (DSHB, B3/D6; 1:200), SOX17 (R&D Systems AF1924; 1:400), ZO-1 (Invotrogen 33–9100; 1:250), pMLC (Cell Signalling 3671; 1:100), Ecad (BD Bioscience 610181; 1:100), Ncad (BD Bioscience 610920; 1:100). For HCRs, the manufacturer’s protocol (Molecular Instruments) was followed. Briefly, embryo tailbuds were dissected from the surrounding extraembryonic tissue and filter paper, fixed for four hours at room temperature in 4% paraformaldehyde in PBS, and rinsed with PBS with 0.5% Triton. Samples were dehydrated with a 25–50-75–100% MeOH (in PBS with 0.5% Triton) gradient (5 min each) and stored overnight at −20C. Then, samples were rehydrated with the reverse MeOH gradient and washed with 0.5% Triton (2×5 min), 50% 0.5% Triton and 50% 5XSSCT (5×2 min) and 5XSSCT (1×5 min). Samples were prehybridized for at least 30 minutes in probe hybridization buffer at 37C and then incubated overnight with probes and buffer at 37C. The next day, samples were washed with pre-warmed probe wash buffer (4× 15 min) and several following washes of 5XSSCT at room temperature (2×5 min, 2× 30 min). Hairpins were snap-cooled (90 seconds at 95C then cooled for 30 minutes in a dark drawer), and the samples were pre-amplified with amplification buffer for at least five minutes. Samples were left covered overnight at room temperature in haripin and amplification buffer solution with DAPI (1:1000). The following day, several 5XSSCT washes (2×5min, 2×30 min, 1×5min), were performed before a few short washes with PBS. Embryos were then cleared in RapiClear (RapiClear 1.49, Sunjin Labs) and mounted on glass slides for imaging.

### Image analysis and Data processing

Image analyses on 3D cleared volumes were performed using the open-source image visualization Python library Napari (Sofroniew et al., 2022), preprocessing functions from the sci-kit image library (van der Walt et al., 2014), the cell segmentation algorithm Cellpose (Stringer et al., 2020).

### Single-cell RNA sequencing

We have optimized a protocol for single cell dissociation from fresh embryonic chick tissues of similar stages, keeping cell viability >90%. Tailbud tissues from embryos were dissected in cold PBS, carefully removing the extraembryonic membranes. To ensure best quality results and tagging efficiency for the downstream computational analysis, we pooled tissues from 12–15 embryos per time group. Cells were enzymatically dissociated in Accutase (STEMCELL Technologies), cell viability and concentration was assessed with a Countess 3 automated cell counter with AO/PI staining. Single-cell libraries were generated using the Chromium GEM-X Single Cell 3’ Reagent Kits v4 (10x Genomics, Pleasanton, CA) according to the manufacturer’s instructions, aiming for 20,000 targeted cell recovery per sample. The final libraries were amplified using dual-index primers (Dual Index Kit TT Set A), then pooled and sequenced on an Illumina NovaSeq X Plus platform to a sequencing depth of approximately 50,000 mean reads per cell.

Raw sequencing data (FASTQ files) were processed using the Cell Ranger 9.0 pipeline (10x Genomics), aligning reads to the Gallus gallus reference transcriptome (GRCg7b), to produce the counts matrix. Downstream analysis was performed using the Scanpy (v1.10+) Python ecosystem. Low-quality cells were excluded based on the following criteria: (1) cells with fewer than 200 or more than 6,000 detected genes; (2) cells with more than 10% of total UMI counts originating from mitochondrial genes; and (3) cells with fewer than 1,000 total UMIs. Doublets were predicted and removed using Scrublet. Gene expression matrices were normalized to 10,000 counts per cell and log-transformed. Highly variable genes (HVGs) were identified using the highly_variable_genes function (flavor=‘seurat’). Principal Component Analysis (PCA) was performed on the scaled data, and the first 50 principal components were used to construct a neighborhood graph. Dimensionality reduction for visualization purposes will use Pairwise Controlled Manifold Approximation (PaCMAP). To define cell populations, we performed unsupervised clustering using the Leiden algorithm. The clustering resolution was optimized (e.g., resolution=0.5) to ensure biological relevance. Differentially expressed genes (DEGs) for each cluster were identified using the rank_genes_groups function in Scanpy, employing the Wilcoxon rank-sum test with Benjamini-Hochberg correction. Genes were considered significant DEGs if they exhibited an adjusted p-value < 0.05 and a log2 fold-change > 0.5. Cluster annotation made use of known markers for the relevant population, leveraging published data from NMP single cell studies (Gouti et al., 2017) and chick datasets (Rito et al., 2025) To investigate the developmental trajectories revealed by the data we employed the CellRank2 package (Weiler et al., 2024) to reconstruct cell state dynamics and infer cellular trajectories. CellRank can flexibly combine various sources of directional information to model cellular state transitions, and by correlating gene expression with computed fate probabilities, it can highlight putative trajectory-specific regulators. We employed the the RealTimeKernel (optimal transport based matching of experimental timepoints) and a precomputed PseudotimeKernel, using the Cytotrace2 potency score, and combined the two kernels in equal weight for the computation of fate probabilities. Subsequently, we defined terminal lineages using the reference macrostates identified from CellRank, and to assemble unique trajectories starting from NE vs NMPs, we subset the data accordingly to compute appropriate lineage absorption scores and respective lineage driver (gene) correlations.

## Supplementary Material

Supplement 1

## Data Availability

scRNAseq data will be deposited on GEO. Code associated with the analyses performed can be found at Github.
