# An endodermal subpopulation gives rise to neuromesodermal progenitors in the posterior chick embryo

**DOI:** 10.64898/2026.05.20.726401

**Published:** 2026-08-05

**Authors:** Panagiotis Oikonomou, Lisa Calvary, Devany Du, Juni Polansky, Giacomo Gattoni, Connor Lynch, Lingting Shi, Christian Mayer, José McFaline-Figueroa, Nandan L. Nerurkar

## Abstract

Embryogenesis occurs through a progressive narrowing of cell fate potential, initiating with the segregation of three distinct germ layers during gastrulation. Although classically, each germ layer contributes to distinct tissue types as development proceeds, this view has been revised with the discovery of neuromesodermal progenitors (NMPs) — a bipotent progenitor population in the posterior embryo that gives rise to traditionally ectodermal and mesodermal tissues after gastrulation has concluded. However, until now the notion of lineage restriction of the endoderm to gastrointestinal, respiratory, and endocrine tissues has largely remained intact. Here, we describe a unique subpopulation in the chick endoderm that initially lines the ventral surface of Hensen’s node (the amniote organizer). As posterior regression of the node ends with termination of the primitive streak, these cells undergo an FGF-dependent epithelial-to-mesenchymal transition, erasing their endodermal identity as they invade the tailbud and subsequently differentiate into a remarkably broad range of cell types including paraxial, lateral plate, and intermediate mesoderm, and to a lesser extent, notochord and neural tube. Disrupting ingression of node endoderm reduced embryonic axis elongation — a process attributed to mesoderm — by 50%. Through lineage barcoding, single-cell RNA sequencing, and fate mapping experiments, we conclude that the endodermal compartment of Hensen’s node harbors a mixed population of fate restricted and multipotent progenitor cells that give rise to clonal populations spanning traditional germ layer boundaries. These findings illustrate a surprising example of germ layer plasticity and fate convergence across distant progenitor populations during amniote development.

## Full Text Availability

The license terms selected by the author(s) for this preprint version do not permit archiving in PMC. The full text is available from the preprint server.

