# Supplementary material for: An endodermal subpopulation generates neural and mesodermal fates in the posterior chick embryo
Source: bioRxiv. 2026 May 22:2026.05.20.726401. Preprint. [Version 1] doi: 10.64898/2026.05.20.726401 (PMC13228638; doi:10.64898/2026.05.20.726401)
Supplement: Supplement 1 [file NIHPP2026.05.20.726401v1-supplement-1.pdf]

bioRxiv preprint doi: https://doi.org/10.64898/2026.05.20.726401; this version posted May 22, 2026. The copyright holder for this preprint (which was not certified by peer review) is the author/funder, who has granted bioRxiv a license to display the preprint in perpetuity. It is made available under a CC-BY 4.0 International license.

SUPPLEMENTARY INFORMATION

An endodermal subpopulation generates neural and mesodermal fates in the posterior chick embryo

Panagiotis Oikonomou[1], Lisa Calvary[1], Devany Du[1], Juni Polansky[1], Giacomo Gattoni[2], Connor Lynch[1], Lingting Shi[1], Christian Mayer[3], José McFaline-Figueroa[1], Nandan L. Nerurkar[1,#]

[1] Department of Biomedical Engineering, Columbia University
[2] Department of Biological Sciences, Columbia University
[3] Max Planck Institute for Biological Intelligence
[#]Correspondence to: **nln2113@columbia.edu**

Supplementary Figures 1
Supplementary Movies 1

bioRxiv preprint doi: https://doi.org/10.64898/2026.05.20.726401; this version posted May 22, 2026. The copyright holder for this preprint (which was not certified by peer review) is the author/funder, who has granted bioRxiv a license to display the preprint in perpetuity. It is made available under a CC-BY 4.0 International license.

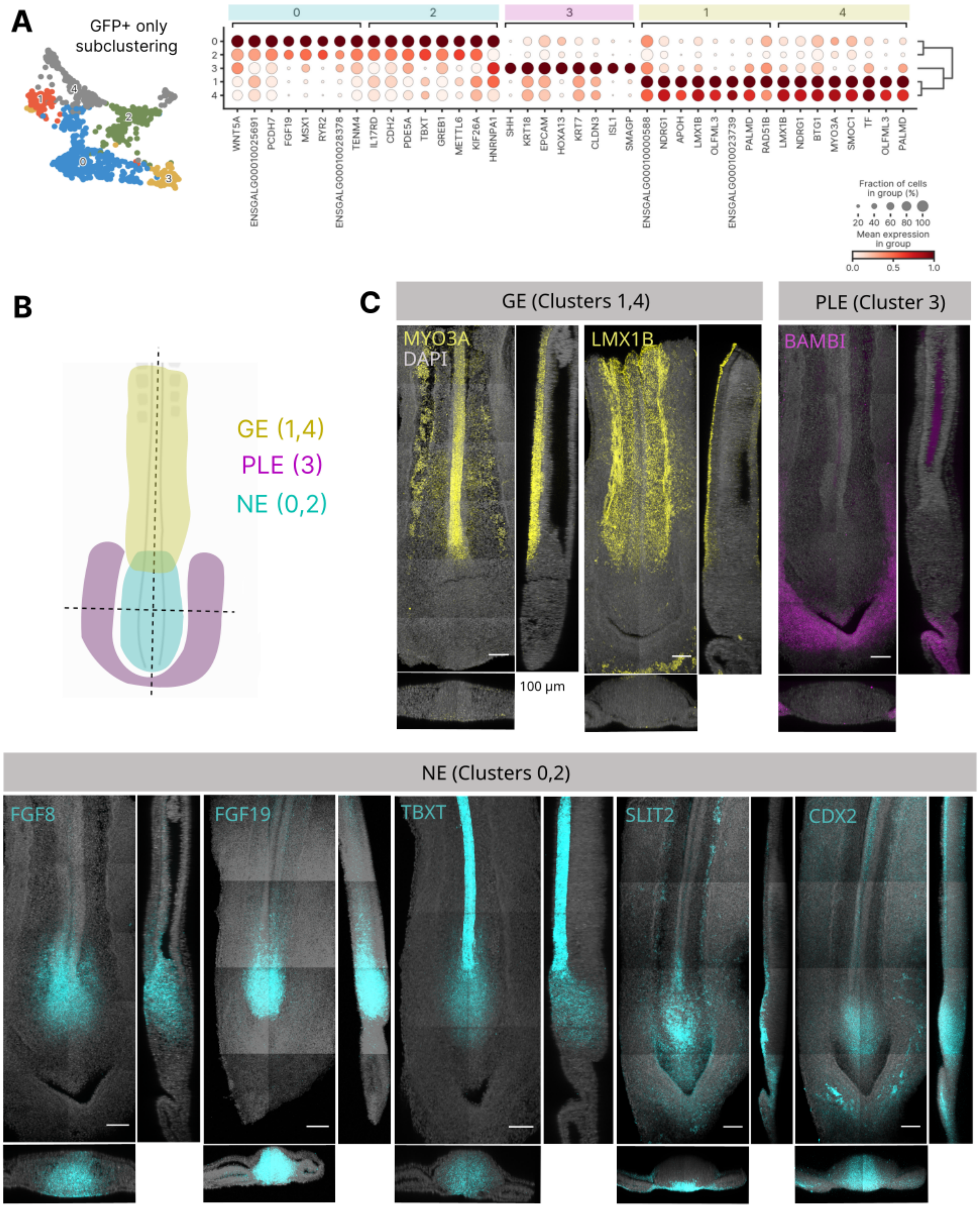

Figure S1. Identification of endodermal subclusters in tagged cells. (**A**) Leiden subclustering and grouping into aggregate clusters. (**B**) Corresponding anatomical regions to clusters in (A). (**C**) HCR validation for gene markers per cluster. GE: Gut endoderm, NE: node endoderm, PLE: postero-lateral endoderm. Saggital views (right) and transverse views (below) are taken along the emmbryonic midline and at the mid-node level, respectively.
